# Muzzling the School Doctor

**Published:** 1912-02-17

**Authors:** 


### "MUZZLING THE SCHOOL DOCTOR."

In our issue of January 27 we published an article under the above heading in which we expressed astonishment that "the fiat had gone forth from the Board of Education that school medical officers, engaged in the inspection of school children, and drawing salaries from local school authorities, are henceforth not to publish any details concerning their work." This statement came from one of the most experienced members of our staff, who has an unusually wide knowledge of school clinics and their work. He is unfortunately absent abroad through illness, which has placed us in a difficulty, seeing that we have heretofore found the information he has supplied accurate and up to date.

In the present instance we regret to state our contributor has evidently made a mistake. Thus we have it on official authority that the statements made in the article referred to as to the attitude of the Board of Education are entirely devoid of substance. We are assured that there is not the least foundation for the suggestion on which the article is based, and that the attitude of this Board has always been exactly the opposite of that which was attributed to it in the article. We have made all possible inquiries and have to express our regret for the error into which our contributor has fallen. Various suggestions have been made as to the causes of the misapprehension which apparently led our contributor to make the statements referred to, but without his personal explanation they must be disregarded.

In making the foregoing correction we may point out that the absolute contradiction which the publication of the original statement has called forth from the highest official sources will be welcomed by the whole medical profession. The school clinic represents a relatively new field of work for practitioners, and to do the maximum of good it is essential that every encouragement shall be given to school medical officers to throw their heart into their work, and to make the teachings and conclusions which are derived from the experience thus gained of the fullest utility to the profession and the public. We are therefore glad to know that not only every school doctor who is on the staff of a hospital, but every school medical officer who is not, can, in fact, deal with his child patients as if they were his hospital patients. We hope each of these officers will acquire the habit of taking complete notes of his cases, and will give the profession the full benefit of his personal report on his experience and work in school clinics. All school medical officers should be greatly encouraged to do this, now they are officially declared to be individually and collectively free to exercise their full professional rights.

We were much interested recently in examining closely a quantity of statistics, which we hope may soon find their way into print, which relate to the diseases to which the children in our public elementary schools are most liable, the diagnosis and treatment of these ailments, and the results of operative interference in a number of cases. We believe that the early publication and wide distribution of the facts of every kind relating to these aspects of the work will speedily result in the establishment of a perfected system of school clinics throughout the country. Now that evidence has accumulated to prove the absolute importance to the nation of a perfected system of examination and treatment of all children in our public elementary schools, the sooner it is placed upon an absolutely satisfactory basis by the provision of an adequate number of school clinics the better will it be for the scholars, the teachers, and the nation.

